# Recurrent Localized Tenosynovial Giant Cell Tumor of the Left Ring Finger: A Case Report and Literature Review

**DOI:** 10.7759/cureus.55962

**Published:** 2024-03-11

**Authors:** Babatope L Awosusi, Omar M Attia

**Affiliations:** 1 Pathology and Laboratory Medicine, King Khalid Hospital, AlMajmaah, SAU; 2 Plastic Surgery, King Khalid Hospital, AlMajmaah, SAU

**Keywords:** left ring finger, recurrent, surgical excision, histopathology, localized, interphalangeal joint, ring finger, tendon sheath, giant cell tumour (gct), tenosynovial

## Abstract

Here, we report the case of recurrent swelling and pain in the proximal interphalangeal joint of the left ring finger, which was later diagnosed as a localized tenosynovial giant cell tumor in a young adult female. The first presentation was at the same anatomical site four years prior. Examination at presentation showed a firm skin-colored nodule in the volar aspect of the left ring finger. The swelling was seen to be partly attached to underlying structures and was non-tender. After a careful physical examination and plain radiograph imaging of the hand, the two differential diagnoses considered were tenosynovial giant cell tumor and ganglion cyst. A surgical excision was performed, and histopathologic evaluation showed features consistent with a tenosynovial giant cell tumor, localized type. The resection margins were clear of tumor. The patient had no intraoperative or postoperative complications. Postoperative physiotherapy was recommended. No recurrence was seen after postoperative surgical follow-up for one year. This report highlights the importance of histopathologic evaluation and confirmation of clear surgical margins in the management of tenosynovial giant cell tumors. In recurrent cases, surgical re-excision with clear margins provides good clinical outcomes. Before surgical excision, patients should be informed about the biologic nature of the lesion and the high risk of recurrence. The management modalities to prevent recurrence and the need for long-term follow-up should also be discussed with the patient.

## Introduction

The most common benign tumor of the hand and wrist region is the ganglion cyst followed by the tenosynovial giant cell tumor (TSGCT) [1]. TSGCT has been described using different terminologies including ''giant cell tumor of the tendon sheath (GCTTS), sclerosing hemangioma, pigmented villonodular synovitis, fibrous xanthoma, and benign synovioma'' [1].

TSGCT is a proliferative lesion affecting synovial joints and tendon sheaths. It usually presents as a firm, painless swelling with a slow progressive growth pattern [2]. TSGCT can either be localized or diffuse with a nodular or multi-lobulated growth pattern. In some cases, TSGCT can also cause impairment of tendon and joint mobility [2]. The localized form of TSGCT is also known as GCTTS [3]. There are two types of TSGCT, namely, intra-articular and extra-articular [4].

The pathogenesis of GCTTS is not clear [4,5]. It can occur at any age but it is most commonly seen in the third to fifth decades of life and it has a female predominance [5]. It has an overall incidence of 1 in 50,000 individuals [4]. TSGCT has a high risk of recurrence, reaching up to 45% according to available literature [2].

Here, we report the case of recurrent localized TSGCT in the distal interphalangeal joint of the left ring finger in a young adult female.

## Case presentation

A 26-year-old female presented at the surgical outpatient department with a history of recurrent swelling in her left ring finger. The first presentation was at the same anatomical site four years prior at a different hospital. The swelling measured 2x1x1 cm then; it was excised with a histopathologic diagnosis of a giant cell tumor of the tendon sheath. Information about the extent of prior surgical resection and margin status was not available at this index presentation.

Examination at this index presentation showed a firm, skin-colored nodule on the volar aspect of the left ring finger at the proximal interphalangeal joint. The swelling was partly attached to underlying structures and was non-tender. There was also associated pain and limitation in movement. Examination of the central and peripheral nervous system was unremarkable. Examination of other systems was also not remarkable.

There was no known chronic illness. There is no significant contributory psycho-social, occupational, or family history. A plain radiograph image of the left hand showed a defined soft tissue mass attached to the tendon sheath of the proximal interphalangeal joint of the ring finger with no bony involvement. After physical and plain radiographic examination of the hand, the two differential diagnoses considered were ganglion cyst and TSGCT.

**Figure 1:**
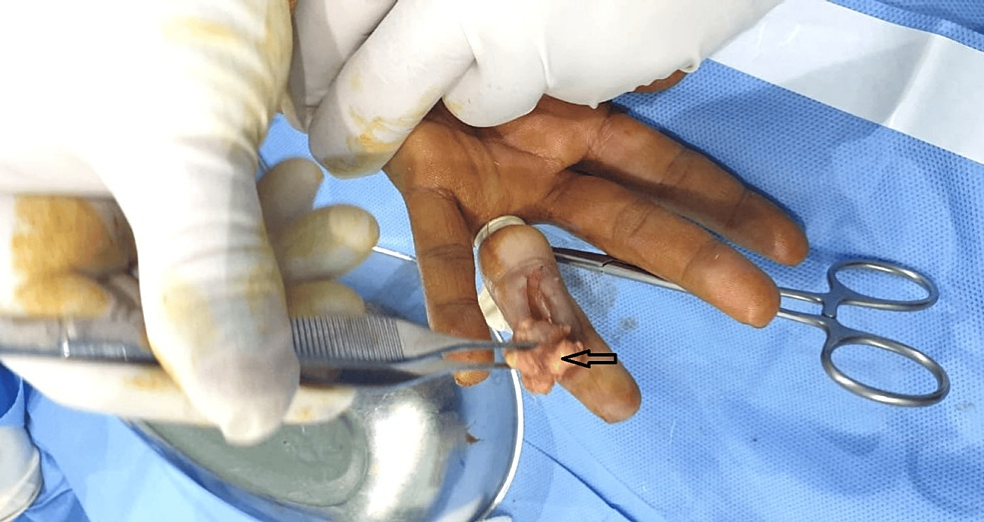
Intraoperative picture showing tumor resection (black arrow) from the left ring finger

A surgical excision was performed (Figure 1), and the sample was sent for histopathologic examination.

**Figure 2:**
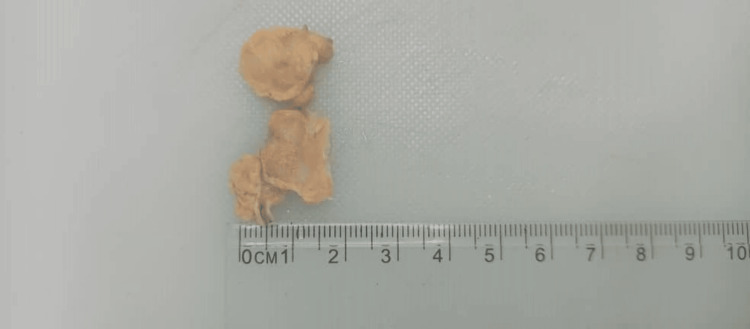
Postoperative picture of the resected specimen

The sample received at surgical grossing was a small, firm, tan tissue weighing 2.8 g and measuring 2.5x1.5x1 cm in dimension. Cut sections showed firm, gray-to-tan surfaces (Figure 2).

**Figure 3:**
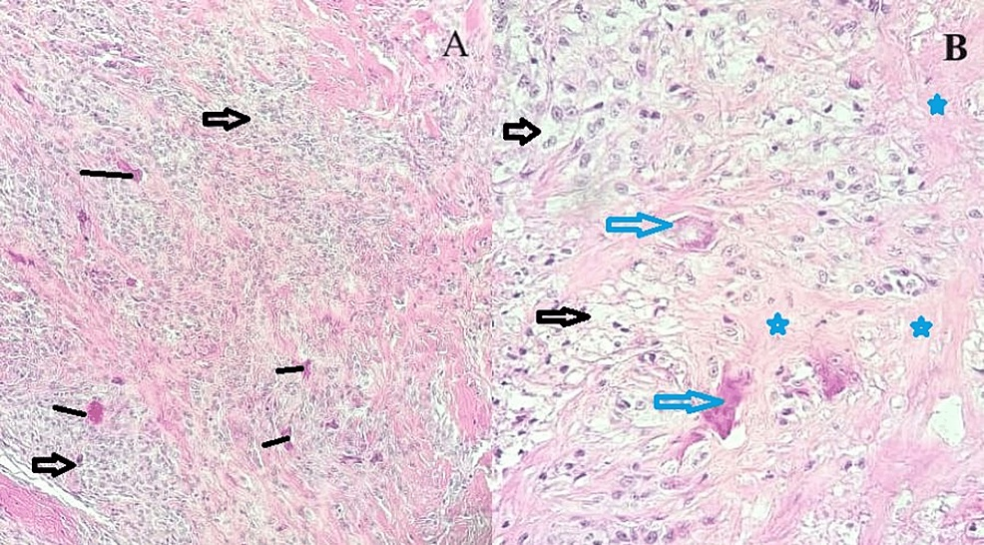
Photomicrograph of the giant cell tumor of the tendon sheath A: x100 magnification; shows a few multinucleated giant cells (black lines) in a background of fibrocollagenous stroma with mononucleated cells (black arrows) B: x100 magnification; shows foamy xanthoma-like cells (black arrows), multinucleated osteoclast-like giant cells (blue arrows), and areas of stromal hyalinization (blue stars)

Microscopic evaluation showed features consistent with a TSGCT, localized type (Figures 3A, 3B). No focus of necrosis or hemorrhage was seen. No focus of atypia or malignant change was seen.

**Figure 4:**
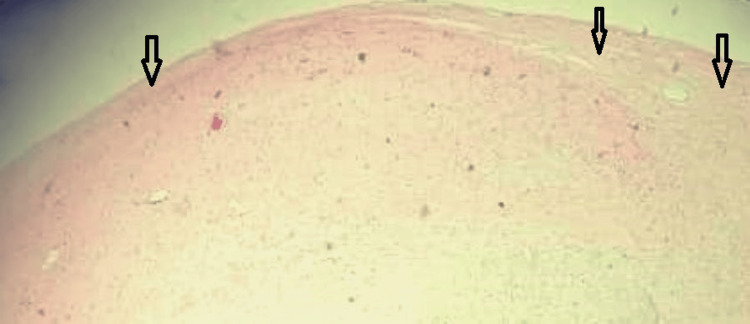
Photomicrograph shows the tumor capsule with variable thickness (black arrows)

Figure 4 shows the fibrous tumor capsule with variable thickness.

The resection margins were clear of tumor. The patient had no intraoperative or postoperative complications. No loss of function or loss of mobility was seen. Postoperative physiotherapy was recommended. No recurrence was seen after surgical follow-up for one year.

## Discussion

TSGCT belongs to a group of proliferative lesions affecting the synovium, bursa, and tendon sheath [5]. Most patients with TSGCT present without symptoms while a subset has impaired joint movement and pain [6]. This is similar to what was seen in this index patient; she presented with swelling, mild pain, and a slight limitation of joint movement. The most affected finger is the index finger in most cases, followed by the middle finger or thumb [1], but in this particular case, the ring finger was affected.

Though the etiopathogenesis of TSGCT is still unknown, the most commonly accepted theory is "reactive or regenerative hyperplasia" with associated chronic inflammation [5] Chromosome 1p13 translocations have been reported in some recent studies [5]. In this particular case, no predisposing factor could be identified. In this index case, the chromosomal analysis was not done because there are no facilities for this investigation at our center.

Detailed history and thorough physical examination are important for accurate diagnosis [6]. GCTTS can cause cortical bone erosions and invasion of the medullary space, which can be seen on plain radiographs [2,6]. Plain radiographs of the hand and wrist show the bony structures and cannot visualize a ganglion cyst as such [7]. Magnetic resonance imaging is a useful diagnostic imaging modality for the classification of GCTTS into Al Qattan types 1 and 2 [6]. An Al Qattan type I tumor is a single round or multi-lobulated lesion while an Al Qattan type 2 tumor represents two or more separate tumors [6]. In this index case, the preoperative diagnosis after careful physical examination and a plain radiograph was that of a ganglion cyst and a giant cell tumor of the tendon sheath. Preoperative MRI was not done so we could not classify it into Al Qattan types.

Complete surgical excision is still the most effective treatment modality for GCTTS [2]. In our index case, after surgical excision, the specimen was sent for histopathologic evaluation, and the diagnosis of GSCTS was confirmed based on histomorphological features. The histology of GCTTS is heterogeneous and consists of multi-nucleated osteoclast-like giant cells, foamy xanthoma-like cells, histiocytes, areas of stromal hyalinization, and varying degrees of hemosiderin and fibrin [8].

The main concern about the management of the lesion is the high recurrence rate [8]. This is similar to what happened in this index patient; the lesion was earlier excised four years prior, and the lesion had now recurred. Incomplete surgical excision is widely accepted as a definitive risk factor [7]. Other known risk factors for local recurrence include the presence of adjacent degenerative joint disease, localization at the distal interphalangeal joint of the fingers or thumb, cortical destruction, type 2 tumors, increased cellularity and mitotic activity, and neurovascular dissection during removal [1,8]. In our patient, we strongly suspect the location at the interphalangeal joint of the finger and incomplete surgical excision in the first instance as the plausible and probable risk factors for recurrence.

Complete surgical excision is the only way to prevent recurrence [8]. There is no strong evidence to support the use of radiotherapy after surgery to reduce the risk of recurrence [8]. Due to this lesion's relatively high risk of recurrence, long-term follow-up is recommended [2]. A follow-up of three years or more may be considered sufficient to rule out future recurrences [6].

## Conclusions

This report highlights the importance of histopathologic evaluation and confirmation of clear surgical margins in the management of tenosynovial giant cell tumors. In cases of recurrent TSGCT, surgical re-excision with clear margins provides good clinical outcomes. Before surgical excision, patients should be informed about the biologic nature of this lesion and the possibility of recurrence. The management modalities to prevent recurrence and the need for long-term postoperative follow-up should also be discussed with the patient.

## References

[1] Çevik HB, Kayahan S, Eceviz E, Gümüştaş SA (2020). Tenosynovial giant cell tumor in the hand: experience with 173 cases. J Hand Surg Asian Pac Vol.

[2] Boeisa AN, Al Khalaf AA (2022). Giant cell tumor of tendon sheath of the distal phalanx. Cureus.

[3] Shergill KK, Pillai HJ, Singh S, Singh R (2023). Giant cell tumor of tendon sheath: a common benign entity with a sore note. Cureus.

[4] Lv Z, Liu J (2020). Giant cell tumor of tendon sheath at the hand: a case report and literature review. Ann Med Surg (Lond).

[5] Chaurasia P (2023). Giant cell tumour of tendon sheath of distal phalanx of thumb: a case report. JNMA J Nepal Med Assoc.

[6] Ozben H, Coskun T (2019). Giant cell tumor of tendon sheath in the hand: analysis of risk factors for recurrence in 50 cases. BMC Musculoskelet Disord.

[7] Żyluk A, Owczarska A (2020). Outcomes of surgery for giant cell tumors of the tendon sheath within the hand. Pol Przegl Chir.

[8] Feger J, Rasuli B, Knipe H (2024). Ganglion cysts of the hand and wrist. Radiopaedia.

